# Medication use and risk of amyotrophic lateral sclerosis—a systematic review

**DOI:** 10.1186/s12916-022-02442-w

**Published:** 2022-08-05

**Authors:** Can Cui, Jiangwei Sun, Kyla A. McKay, Caroline Ingre, Fang Fang

**Affiliations:** 1 Unit of Integrative Epidemiology, Institute of Environmental Medicine, Karolinska Institutet, Stockholm, Sweden; 2 Department of Clinical Neuroscience, Karolinska Institutet, Stockholm, Sweden; 3 Centre for Molecular Medicine, Karolinska Institutet, Stockholm, Sweden; 4 Neurology Clinic, Karolinska University Hospital, Stockholm, Sweden

**Keywords:** Medication, Amyotrophic lateral sclerosis, Risk, Association

## Abstract

**Background:**

Studying whether medications act as potential risk factors for amyotrophic lateral sclerosis (ALS) can contribute to the understanding of disease etiology as well as the identification of novel therapeutic targets. Therefore, we conducted a systematic review to summarize the existing evidence on the association between medication use and the subsequent ALS risk.

**Methods:**

A systematic review was conducted in Medline, Embase, and Web of Science from the date of database establishment to December 10, 2021. References of identified articles were further searched for additional relevant articles. Studies were included if (1) published in English, (2) explored medication use as exposure and development of ALS as outcome, and (3) the design was a human observational study. Clinical trials, reviews, comments, editorials, and case reports were excluded. Quality assessment was performed using a pre-validated tool for non-randomized studies, the Newcastle–Ottawa Assessment Scale (NOS).

**Results:**

Of the 4760 studies identified, 25 articles, including 13 case–control studies, five nested case–control studies, six cohort studies, and one retrospective chart review, were included in the review. Among these studies, there were 22 distinct study populations that included 171,407 patients with ALS, seven classes of medication examined, and 23 studies with a NOS ≥ 5. There was a general lack of agreement between studies on the associations of cholesterol-lowering drugs, anti-inflammatory drugs, immunosuppressants, antibiotics, oral contraceptives (OCs) or hormone replacement therapy (HRT), antihypertensive drugs, antidiabetics, and drugs for psychiatric and neurological disorders with the subsequent risk of ALS. However, it appeared that statins, aspirin, OCs/HRT, antihypertensives, and antidiabetics were unlikely related to a higher risk of ALS. The positive associations noted for antibiotics, antidepressants, and skeletal muscle relaxants might be attributable to prodromal symptoms of ALS.

**Conclusions:**

There is currently no strong evidence to link any medication use with ALS risk.

**Supplementary Information:**

The online version contains supplementary material available at 10.1186/s12916-022-02442-w.

## Background

Amyotrophic lateral sclerosis (ALS) is a neurodegenerative disease, featuring progressive loss of motor neurons in the brain and spinal cord. Globally, around 4.5 per 100,000 people are living with this disease and 1.9 per 100,000 people are newly diagnosed with ALS each year [1]. As no effective treatment is currently available, patients with ALS usually die within 3–5 years after symptom onset [2]. The cause of ALS remains unknown. Although various risk factors have been proposed, older age, male gender, family history, and specific genetic mutations are the only established risk factors [3]. A vivid area of research in ALS concerns medication use, with the aim to improve understanding of disease etiology as well as identify novel therapeutic targets (e.g., through drug repurposing). To this end, we performed a systematic review of the existing literature aiming to provide a comprehensive view of the current knowledge on the roles of different medication use on the subsequent risk of ALS.

## Methods

This systematic review was conducted according to the Preferred Reporting of Systematic Reviews and Meta Analyses (PRISMA) guidelines [4]. A unique searching strategy (online material) was applied in the databases Medline (1947-), Embase (1974-), and Web of Science (1900-) from the dates of database establishment to December 10, 2021. References of identified articles were further searched for additional relevant articles.

### Inclusion and exclusion criteria

All original articles in English which used medication use as exposure and development of ALS/ motor neuron disease (MND) as outcome among human subjects were included. Clinical trials, systematic reviews and meta-analyses, comments, editorials, and case reports were excluded.

### Ascertainment of exposure and outcome

Exposure was defined as any medication use according to questionnaire data, medical records, or drug prescription registers. Alternative medicine which lacks biological plausibility and is untested or dietary supplements were not considered as exposure. The diagnosis of ALS was derived from patient register, medical records, or other sources.

### Study selection and quality assessment

The screening process was divided into two stages (stage 1: title and abstract; stage 2: full text). Two researchers independently evaluated the articles and reached consensus at each stage. The screening tools Rayyan and Endnote were used. A pre-validated tool, the Newcastle–Ottawa Assessment Scale (NOS) [5], was used at the final stage for quality assessment of included articles. The NOS scales the quality of each article based on the selection of study groups, the comparability of the groups, and the ascertainment of the exposure and outcome of interest (from 0 to 9; 9 being the highest quality).

## Results

**Fig. 1:**
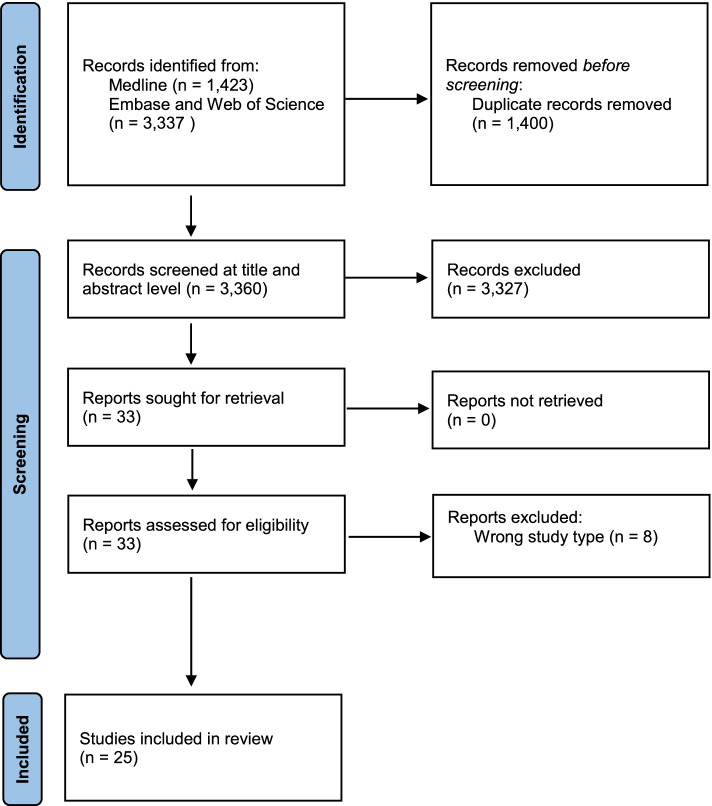
Flowchart of the study selection. Steps and number of articles included and excluded per step during the study selection process

Out of a total 3360 unique studies that were screened by title and abstract, 33 articles were selected for a full-text screening. After consensus by the two researchers who performed the screening independently, 25 articles were included in the review (Fig. 1). Many articles were excluded because of wrong article type (review, clinical trial, post-marketing surveillance, etc.), exposure (dietary supplements, chemicals, etc.), outcome (prognosis, comorbidities, etc.), or study subject (mice, cell cultures, etc.). Finally, a total of seven categories of medications, including cholesterol lowering drugs, anti-inflammatory drugs, immunosuppressants, antibiotics, oral contraceptives (OCs) or hormone replacement therapy (HRT), antihypertensive drugs, antidiabetics, and drugs for psychiatric and neurological disorders, were assessed for an association with risk of ALS.

Table 1 summarizes the characteristics of the 25 articles, including publication information, study type, demographics of the participants, data source, exposure, and outcome ascertainment, adjusted covariates, main findings, consideration of reverse causation, and quality score. Among these studies, there were 22 distinct study populations including 171,407 persons with ALS. Nineteen of these studies considered to some extent reverse causation and 23 studies had a quality score ≥ 5.

**Table 1:** Literature summaries of association between medication use and risk of amyotrophic lateral sclerosis (25 articles including 7 drug categories)

Author (year), country	Study design	No. of cases/controls or exposed/unexposed participants	Mean follow-up time (years)	Mean age (years)	Male (%)	Source of participants	Exposure	Definition of medication use (ATC code); way of comparison	Ascertainment of ALS	Main findings	Adjusted covariates	Consideration of reverse causation; influence on main findings	Quality score
Pfeiffer et al. [6], USA	Population-based case–control study	10,450/ 104,500	–	74	49	US Medicare beneficiaries	685 prescription drugs and a priori hypothesized sex hormone drugs	Register-based data (ATC not available); ever/never	Register-based data	Lower ALS risk in relation to azithromycin (OR = 0.79), amlodipine (OR = 0.85), furosemide (OR = 0.75), lisinopril (OR = 0.86), metoprolol (OR = 0.84), digoxin (OR = 0.69), warfarin (OR = 0.79), human recombinant glucagon (OR = 0.75), potassium chloride (OR = 0.8) and tamoxifen (OR = 0.61). Higher ALS risk in relation to baclofen (OR = 2.21) and testosterone (OR = 4.92)	Age, sex, calendar year, race, indicators of socio-economic status, Medicare use, selected comorbidities, and physician visits per 6-month period	Considered by using 1-year and 3-year lag times; no difference	8
Sorensen et al. [7], Denmark	Population-based case–control study	556/5560	–	66	54%	Danish national registers of hospital visits and drug prescription northern Denmark	Statins	Register-based data (C10AA); ever/never; recent/former/never; duration of use	Register-based data	No association between statin and risk of ALS (OR = 0.96; 95% CI 0.73–1.28)	Sex, birth year, calendar time, and medical indications	Considered by using 1–59, > = 60 days; before ALS diagnosis; similar	9
Sutedja et al. [8], The Netherlands	Case–control study	334/538	–	60	57	University Medical Centre Utrecht	Cholesterol lowering drugs, antihypertensives, and antidiabetics	Questionnaire-based data (ATC not available); ever/never	Hospital-based data	Lower risk of ALS in relation to cholesterol lowering agents (OR = 0.6; 95% CI 0.4–0.9). No association for antihypertensives or antidiabetics	Age and sex	Considered by extracting drug use prior to disease onset; no difference	4
Seelen et al. [9], The Netherlands	Population-based case–control study	722/2268	–	63	60	Prospective ALS study in the Netherlands (PAN)	Statins and immunosuppressive drugs	Questionnaire-based data (ATC not available); ever/never	Hospital-based, register-based, and web-based data	Lower risk of ALS in relation to statins (OR = 0.45, 95% CI 0.35–0.59) or immunosuppressive drugs (OR = 0.26, 95% CI 0.08–0.86)	Gender, age, education, current smoking, and current alcohol consumption	Considered by extracting drug use prior to disease onset; no difference	7
Freedman et al. [10], USA	Nation-wide population-based case–control study	10,450/104,500	–	74	49	U.S. Medicare beneficiaries	Statins and other cholesterol-lowering drugs	Register-based data (ATC not available); ever/never; duration of use	Register-based data	Lower ALS risk in relation to statins (OR = 0.87, 95% CI 0.83–0.91), but not other cholesterol lowering drugs (nitrates, bile acid sequestrants, and ezetimibe)	Age, sex, and calendar year, race, socioeconomic status, Medicare use, indications for statin prescription, obesity, and chronic obstructive pulmonary disease as surrogate for smoking, average number of physician visits per 6-month period	Considered by using 1-year and 3-year lag times; no difference	8
Torrandell-Haro et al. [11], USA	Retrospective cohort study	144,214/144,301	5.1	67 for individuals diagnosed with ALS and 66 for others	47 for individuals diagnosed with ALS and 43 for others	Humana database	Statins	Register-based data (ATC not available); ever/never	Register-based data	Statin use was associated with a lower risk of ALS (RR = 0.46; 95% CI 0.30–0.69)	Propensity score matched exposed and unexposed individuals, considering age, sex, race, region, type 2 diabetes, hypertension, cardiovascular disease, and cerebrovascular disease	Considered by using 1-year lag time; no difference	8
Mariosa et al. [12], Sweden	Population-based nested case–control study	2,475/12,375	–	68 for male and 70 for female	57	Swedish Total Population Register, Swedish Patient Register, and Swedish Prescribed Drug Register	Antidiabetics and statins	Register-based data (antidiabetics: A10; statins: C10AA)	Register-based data	Lower ALS risk in relation to antidiabetics use (OR = 0.76; 95% CI 0.65–0.90). No association for statin use (OR = 1.08; 95% CI 0.98–1.19)	Matching factors (age, sex and area of residence)	Considered by using < 1, 1–2, 2–3, 3–4, 4–5, or 5–8 years before ALS diagnosis; strong effect on statin	8
Skajaa et al. [13], Denmark	Population-based cohort study	974,304/1,948,606	7.7	63y for both statin users and non-users	52% for both statin users and non-users	Danish National Patient Registry, and Danish National Prescription Registry	Statins	Register-based data (ATC not available)	Register-based data	A weak association between statin and risk of ALS in general (OR = 1.11; 95% CI 1.00–1.23), especially among women (1.29 (95% CI 1.11–1.50)	Sex, birth year, calendar year, medically diagnosed comorbidities, and concomitant medications	Considered by using time windows 0–1, > 1–5, > 5–10, and > 10–22 years after drug use; strong effect	9
Popat et al. [14], USA	Case–control study	111/258	–	63 for cases and 62 for controls	59 for cases and 62 for controls	Kaiser Permanente Medical Care Program of Northern California	Non-steroidal anti-inflammatory drugs (NSAIDs)	Interview-based data (ATC not available); ever/never; current/former/never; duration of use	Register-based and hospital-based data	No association between ALS and non-aspirin NSAID use (OR = 1.1; 95% CI 0.7–1.9) or aspirin use (OR = 1.1; 95% CI 0.7–1.8)	Age, gender, history of osteoarthritis/rheumatoid arthritis and pain, and other medication use	Not considered	5
Fondell et al. [15], USA	Prospective cohort study	708 cases of ALS among 9,727,583 person-years contributed by 786,274 participants of different cohorts	15	–	–	Nurses’ Health Study, Health Professionals Follow-up Study, Cancer Prevention Study II Nutrition Cohort, Multiethnic Cohort Study, and National Institutes of Health–AARP Diet and Health Study	NSAIDs	Questionnaire-based data (ATC not available); ever/never	Register-based, self-reported, and medical record data	No association for non-aspirin NSAID use (RR = 0.96; 95% CI 0.76–1.22) or aspirin use (RR = 1.07; 95% CI 0.92–1.25)	Smoking status, educational level, body mass index (BMI), physical activity level, use of vitamin E supplements	Considered by using 4-year lag time; similar	5
Tsai et al. [16], Taiwan	Population-based case–control study	729/7290	–	57 for cases and controls	62 for cases and controls	The National Health Insurance Research Database	Aspirin	Register-based data (N02BA01); ever/never, and cumulative defined daily dose	Register-based and medical record data	Lower ALS risk in relation to aspirin use (OR = 0.69; 95% CI 0.56–0.87)	Sex, age, residence, insurance premium, use of diphenhydramine, mefenamic acid, and steroid	Considered by excluding drug use in the year prior to diagnosis; similar; no difference	8
Kuczmarski et al. [17], USA	Case–control study	414/361	–	61 for cases and controls	63 for cases and 59 for controls	Department of Neurology at Dartmouth–Hitchcock Medical Center, Lebanon, New Hampshire, the Department of Neurological Sciences at the University of Vermont Medical Center, Burlington, Vermont, and the Department of Neurology at Johns Hopkins Medicine in Baltimore, Maryland	Chemotherapy or immunosuppressive drugs	Questionnaire-based data (ATC not available); ever/never	Hospital-based data	Lower ALS risk in relation to chemotherapy (OR = 0.46, 95% CI 0.22–0.89). No association for immunosuppressant use (OR = 0.78, 95% CI 0.50–1.02)	Age, gender, and smoking	Not considered	4
Sun et al. [18], Sweden	Population-based nested case–control study	2,484/12,420	–	69 for cases and controls	57 for cases and controls	Swedish Patient Register, Total Population Register, and Prescribed Drug Register	Antibiotics	Register-based data (J01A-J01X); ever/never; frequency of use	Register-based data	A dose–response association between antibiotics use and risk of ALS was found. Higher risk of ALS in relation to antibiotics use (OR = 1.11, 95% CI 1.01–1.22.)	Age, sex, and area of residence	Considered by using 1–3-year lag times; similar	7
Popat et al. [19], USA	Case–control study	62/131	–	68 for cases and controls	0	Kaiser Permanente Medical Care Program of Northern California	Postmenopausal hormone or oral contraceptive use	Interview-based data (ATC not available); ever/never; current/former/never; duration of use	Register-based and hospital-based data	No association for postmenopausal hormone use (OR = 1.9, 95% CI 0.9–3.8) or oral contraceptive use (OR = 0.6, 95% CI 0.3–1.6)	Age, respondent type, type of menopause (postmenopausal hormone use) and NSAID use	Considered by using 5-year lag time; similar	5
Doyle et al. [20], UK	Prospective cohort study	752 MND cases of among 1,319,360 women	9.2	–	0	The Million Women Study	Oral contraceptives (OCs) or hormone replacement therapy (HRT)	Questionnaire-based data (ATC not available); ever/never; current/past/never; duration of use	Register-based data	No association for OCs or HRT	Year of birth, region, deprivation, smoking, alcohol use, HRT use, and BMI	Not considered	6
Rooney et al. [21], Ireland, Italy, and the Netherlands	Case–control study	653/1,217	–	64–67	0	Euro-MOTOR study	OCs or HRT	Questionnaire-based data (ATC not available); ever/never, duration of use	Register-based and hospital-based data	Lower ALS risk in relation to OCs (OR = 0.65, 95%CI 0.51–0.84) with a dose–response effect in all three countries. Lower risk in relation to HRT (OR = 0.57, 95% CI 0.37–0.85) in the Netherlands	Age, education, study site	Considered by using excluding drug use 3 years prior to diagnosis; no difference	5
Diekmann et al. [22], Germany	Case–control study	200/197	–	62 for cases and 59 for controls	64 for cases and 56 for controls	Hannover Medical School	Contraceptives, magnesium, antidepressants, NSAIDs, statins, thyroid medication, antidiabetics, steroids, immunosuppressants, neuroleptics, or homeopathic medicine	Questionnaire-based data (ATC not available); ever/never	Hospital-based data	Higher ALS risk in relation to magnesium (OR = 2.8, 95% CI 1.4–5.8), antidepressants (OR = 3.2, 95% CI 1.6–6.6) and NSAIDs (OR = 2.3, 95% CI 1.2–4.4). A lower ALS risk in relation to contraceptives (OR = 0.4, 95% CI 0.2–0.7). No association for statins, thyroid medication, antidiabetics, steroids, immunosuppressants, neuroleptics, or homeopathic medicine	Age, gender, BMI, occupation, and physical activity	Not considered	5
Kim et al. [23], USA	Retrospective cohort study	189,676/189,676	5.1	68y for exposed and 67.5y for unexposed	0	Humana database	HRT	Register-based data (ATC not available)	Register-based data	Lower ALS risk in relation to HRT (RR = 0.42, 95% CI 0.28–0.63, *P* < 0.001) and oral HRT (RR = 0.40, 95% CI 0.26–0.61, *P* < 0.001)	Propensity score-based matching between exposed and unexposed individuals, including age, race, comorbidities, and Charlson Comorbidity Index (CCI)	Considered by using 1-year lag time; no difference	8
Lin et al. [24], Taiwan	Population-based case–control Study	729/14,580	–	57 for cases and controls	62 for cases and controls	National Health Insurance	Angiotensin-converting enzyme inhibitors (ACEIs), aspirin, antihypertensives, steroids, and NSAIDs	Register-based data (ATC not available); ever/never; cumulative defined daily dose (cDDD)	Register-based and medical record data	Dose-dependent lower ALS risk in relation to ACEI (for < 449.5 cDDD: OR = 0.83, 95% CI 0.65–1.07; for > 449.5 cDDD, OR = 0.43, 95% CI 0.26–0.72; for any use: OR = 0.74, 95% CI 0.58–0.94) and aspirin. No association for other antihypertensives, steroids, or NSAIDs	Sex, age, residence, insurance premium, other antihypertensives, aspirin, steroids, NSAIDs, CCI, length of hospital stay, and number of outpatient visits	Considered by using excluding drug use 1 year prior to diagnosis; no difference	8
Franchi et al. [25], Italy	Population-based nested case–control study	1200/120,000	–	68 for cases and controls	56 for cases and controls	Administrative database of the Lombardy Region, Northern Italy	ACEIs, angiotensin II receptors blockers (ARBs)	Register-based data (ATC not available); ever/never; cDDD	Register-based and medical record data	No association for ACEIs or ARBs	Gender, age, and area of residence	Considered by using excluding drug use 1 year prior to diagnosis; no difference	5
Cetin et al. [26], Sweden	Population-based nested case–control study	2484/24,840	–	69 for cases and controls	57 for cases and controls	Swedish Total Population Register, Swedish Patient Register, Swedish Prescribed Drug Register, and Causes of Death Register	Proton pump inhibitor (PPIs)	Register-based data (A02BC); ever/never; cDDD	Register-based data	No association (OR = 1.08, 95% CI 0.97–1.19) for PPI use when utilizing a lag window of at least 1 year before diagnosis	Sex, age, and area of residence	Considered by using 1–3-year lag times; no difference	7
Garwood et al. [27], USA	Case–control study	72/58	–	53 for cases	63 for cases and 33 for controls	University of California, San Francisco Neurology Faculty clinics	Amphetamine	Questionnaire-based data (ATC not available); ever/never	Hospital-based data	No association for amphetamine use (OR = 2.75, 95% CI 0.6–12.0)	Caffeine, tobacco use, alcohol, age, and gender	Not considered	5
Roos et al. [28], Sweden	Population-based nested case–control study	1752/8760	–	66–75 for cases and controls	57 for cases and controls	Swedish Patient Register, Swedish Prescribed Drug Register, Causes of Death Register, and Swedish Education Register	Antidepressants	Register-based data (N06AA, N06AB, N06AG and N06AX); ever/never	Register-based data	Higher ALS risk in relation to antidepressant use (1 year before diagnosis: OR = 5.8, 95% CI 4.5–7.5; 1–2 year before diagnosis: OR = 1.9, 95% CI 1.4–2.5; > 3 years before diagnosis: OR = 1.4, 95% CI 1.1–1.7)	Year of birth, sex, region of residence, educational level, and socioeconomic status	Considered by using < 1, 1–2, 2–3, > 3 years before ALS diagnosis; strong effect	7
Prosser et al. [29], USA	Retrospective chart review	577/451	–	41 for patients receiving Lithium and 43 for patients not receiving Lithium	46	The Lithium Archive Project	Lithium	Medical record data (ATC not available); ever/never	Medical record data	A lower ALS risk in relation to lithium (OR = 0.1, 95% CI 0.01–0.92)	Age, duration of clinic attendance, and use of anti-psychotic medications	Not considered	6
D’Ovidio et al. [30], Italy	Prospective cohort study	300/687,024	–	60–74 for cases and 45–59 for controls	55 for cases and 47 for controls	2001 census data, Municipality Registry, the Anatomical Therapeutic Chemical Drug Prescription Registry, the Piedmont and Valle d’Aosta ALS Register (PARALS), and Piedmont Regional Drugs Registry	Opioids, antiepileptic drugs, anti-parkinsonian drugs, antipsychotics, and antidepressants	Register-based data (opioids: N02A, antiepileptic drugs: N03A, anti-parkinsonian drugs: N04, antipsychotics: N05A, and antidepressants: N06A); ever/never; and categorical cumulative dose	Register-based data	A lower ALS risk in relation to opioids (HR = 0.59, 95% CI 0.35–0.97) and a marginal higher risk in relation to antiepileptics (HR 1.35, 95% CI 0.92–2.00). No association for anti-parkinsonian drugs, antipsychotics, and antidepressants	Sex, age, education, marital status, and drug co-exposure	Considered by using 1-, 2-, and 5-year lag times: similar	8

*ACEIs* Angiotensin-converting enzyme inhibitors, *ALS* Amyotrophic lateral sclerosis, *ARBs* Angiotensin II receptors blockers, *BMI* Body mass index, *cDDD* Cumulative defined daily dose, *CI* Confidence interval, *HR* Hazard ratio, *HRT* hoRmone replacement therapy, *NSAIDs* Non-steroidal anti-inflammatory drugs, *OCs* Oral contraceptives, *OR* Odds ratio, *PAN* Prospective ALS study in the Netherlands, *PARALS* The Piedmont and Valle d’Aosta ALS Register, *PPIs* Proton pump inhibitor, *RR* Relative risk

### Cholesterol lowering drugs (mainly statins)

Eight studies have explored the associations of cholesterol lowering drugs with the subsequent risk of ALS [7–13, 22], among which seven studies focused on statins [7, 9–13, 22] while one focused on a collection of cholesterol lowering drugs [8]. Four studies demonstrated a protective role of cholesterol lowering drugs in ALS development [8–11], while another four reported a lack of or near-null association between statins and risk of ALS [7, 12, 13, 22].

A case–control study from the Netherlands included 334 patients with ALS and 538 age- and sex- matched controls and found an inverse association between cholesterol lowering agent use and ALS risk (OR = 0.6; 95% CI 0.4–0.9) [8]. A US population-based case–control study included 10,450 cases and 104,500 age-, sex-, and calendar year-matched controls and found a 13% lower ALS risk in relation to statin use, but not for non-statin anti-cholesterol medication use, after adjusting for age, sex, calendar year, race, socioeconomic status, Medicare use, indications for statin prescription, obesity, chronic obstructive pulmonary disease (COPD), and average number of physician visits [10]. In this study, lipophilic statins showed greater inverse association with ALS, compared with other statins [10]. Another Dutch study also found a 55% lower risk of ALS in relation to statin use in a case–control study of 722 patients with sporadic ALS and 2268 age- and gender-matched controls, adjusted for gender, age, education, current smoking, and current alcohol consumption [9]. Excluding smoking and alcohol consumption or adding BMI in the multivariate analyses or excluding patients with *C9orf72* repeat expansion did not change the result [9]. Finally, a US retrospective cohort study, including 144,214 individuals with statin use and 144,301 individuals without statin use, who were individually matched by a propensity score, found statin use to be associated with a lower risk of ALS (RR = 0.46; 95% CI 0.30–0.69) [11].

In contrast, four studies showed a null or near-null association between statin use and risk of ALS, including a Danish population-based case–control study with 556 cases and 5560 controls [7], a Swedish nested case–control study with 2475 cases and 12,375 controls [12], and a German case–control study with 200 cases and 197 controls [22], demonstrating a null association; and a Danish population-based cohort study including 974,304 individuals with statin use and 1,948,606 individuals without statin use [13], showing a weak association between statin use and ALS risk.

### Anti-inflammatory drugs, immunosuppressants, or antibiotics

Two studies examined steroids [22, 24] and five have evaluated the relationship of non-steroidal anti-inflammatory drugs (NSAIDs) [14–16, 22, 24] in relation to risk of ALS, among which four studies examined aspirin [14–16, 24]. In addition, three studies assessed the association between immunosuppressant use as a broad category and ALS risk [9, 17, 22] whereas two studies explored antibiotic use in relation to ALS risk [6, 18], focusing on a broad category of drugs or sub-class or individual drugs.

The findings on the association of NSAIDs with ALS risk are not conclusive. A German case–control study found a twofold risk increase of ALS in individuals with use of NSAIDs (OR = 2.3, 95% CI 1.2–4.4) [22]. However, two US studies, including one case–control study (111 cases and 258 controls) and one prospective cohort study (708 cases of ALS during a follow-up of 9,727,583 person-years) found that neither non-aspirin NSAID nor aspirin use was associated with the risk of ALS [14, 15]. A population-based case–control study in Taiwan including 729 ALS cases and 14,580 controls also failed to observe an association between NSAID use and risk of ALS [24]. However, together with another population-based case–control study in Taiwan (including the same 729 cases as the previously mentioned study in Taiwan and 7290 controls) [16], both studies showed a lower ALS risk with aspirin use, including some evidence for a dose–response relationship [16]. When it comes to steroids, no association with ALS was found in either the German or Taiwanese study [22, 24].

Regarding immunosuppressants, a Dutch population-based case–control study found a 74% reduced ALS risk in relation to the use of immunosuppressive drugs (OR = 0.26, 95% CI 0.08–0.86) [9]. A US case–control study [17] and a German case–control study [22] failed however to observe an association between immunosuppressant use and ALS. The US study did report a lower ALS risk among individuals with a history of chemotherapy (OR = 0.46, 95% CI 0.22–0.89) [17].

A nested case–control study in Sweden, including 2484 newly diagnosed patients with ALS and 12,420 sex-, birth year-, and area of residence-matched population controls, found that antibiotic use was associated with a higher risk of ALS (OR = 1.11, 95% CI 1.01–1.22) with a dose–response relationship (*P* for trend = 0.0069), while no association was noted for macrolide-type antibiotics [18]. A US study found azithromycin, a macrolide-type antibiotic, to be associated with a lower risk of ALS, on the other hand [6].

### OCs or HRT

Five studies have assessed the roles of OCs or hormone use as a broad category on the risk of ALS [19–23]. No association was found between OC or HRT use and ALS risk in either a US case–control study with 62 cases and 131 controls [19] or a British prospective cohort study including 1,319,360 women among whom 752 developed MND [20]. However, a case–control study conducted in Ireland, Italy, and the Netherlands found a lower risk of ALS in relation to OC use (OR = 0.65, 95% CI 0.51–0.84) with a dose–response relationship in all three countries, whereas a lower risk in relation to HRT use was only shown in the Netherlands (OR = 0.57, 95% CI 0.37–0.85) [21]. In addition, OC use was found to be associated with a lower risk of ALS (OR = 0.4, 95% CI 0.2–0.7) in a German case–control study [22], and HRT use was found to be associated with a lower ALS risk in a US cohort study (RR = 0.42, 95% CI 0.28–0.63) [23].

### Antihypertensive drugs

Four studies have evaluated the association of antihypertensive use with risk of ALS [6, 8, 16, 25]. A case–control study in the Netherlands did not find an association between any antihypertensive use and ALS risk [8]. A US population-based case–control study, on the other hand, screened 685 individual prescription drugs and found that, among antihypertensive drugs, the use of amlodipine (calcium channel blocker), furosemide (loop diuretic), lisinopril (angiotensin-converting enzyme inhibitor/ACEI), or metoprolol (beta blocker) was associated with a lower risk of ALS [6]. Two other studies examined the roles of ACEIs or angiotensin II receptor blockers (ARBs) on ALS risk. The first is an Italian nested case–control study, with 1200 cases and 120,000 controls, showing no clear association between ACEIs or ARBs and the risk of MND [25]. In contrast, the other study, a case–control study in Taiwan with 729 cases and 14,580 controls found use of ACEIs to be associated with a lower risk of ALS, including a dose–response relationship when studying ACEIs by cumulative defined daily dose (cDDD) [16]. However, no association was found for other antihypertensives [16]. Among the individual compounds of ACEIs, captopril and enalapril were shown to be associated with a lower ALS risk [16].

### Antidiabetics

Four studies have estimated the association of antidiabetic use with the risk of ALS [6, 8, 12, 22]. Among these, two case–control studies (one from the Netherlands and one from Germany) did not observe an association between antidiabetic use and risk of ALS [8, 22]. Contrary to these findings, a nested case–control study in Sweden reported a lower risk of ALS among persons exposed to antidiabetics, among which an inverse association was noted for insulin, metformin and sulfonylureas [12]. A US study also found a lower ALS risk among individuals who used human recombinant Glucagon using either a 1- or 3-year lag time, although a lower risk for metformin was only noted using a 1-year lag time, compared with controls [6].

### Proton pump inhibitor (PPI)

Only one study has evaluated the association between PPI use and ALS risk [26]. This study included 2484 cases with ALS and 24,840 controls in Sweden and failed to find an association between PPI use and ALS risk [26].

### Drugs for psychiatric and neurological disorders

An Italian prospective cohort study with 687,324 participants examined the association of multiple nervous system drugs (opioids, antiepileptic drugs, anti-parkinsonian drugs, antipsychotics, and antidepressants) with ALS risk [30]. In this study, a 41% lower ALS risk was found in relation to the use of opioids (HR = 0.59, 95% CI 0.35–0.97) and a higher ALS risk was suggested in relation to the use of antiepileptics (HR 1.35, 95% CI 0.92–2.00), whereas no association was noted for use of anti-parkinsonian drugs, antipsychotics, or antidepressants [30]. A nested case–control study in Sweden found however a higher prevalence of antidepressant use before ALS diagnosis, compared with controls [28]. A German case–control study found a threefold risk increase of ALS in individuals with use of antidepressants (OR = 3.2, 95% CI 1.6–6.6) [22]. Lithium, used for bipolar and some depressive disorders, was found to be associated with a lower ALS risk in a US cohort study [29]. The central nervous system (CNS) stimulant amphetamine, used in the treatment of attention deficit hyperactivity disorder (ADHD) and narcolepsy, was not found to be associated with ALS risk in a US case–control study [27]. Skeletal muscle relaxant, baclofen, was found to be associated with a higher risk of ALS in a US case–control study [6].

## Discussion

This systematic review summarized all available studies assessing the link between the use of seven classes of medications, including cholesterol lowering drugs, anti-inflammatory drugs, immunosuppressants, and antibiotics, oral contraceptives (OCs) or hormone replacement therapy (HRT), antihypertensive drugs, antidiabetics, proton pump inhibitors, and drugs for psychiatric and neurological disorders and risk of ALS. In brief, we found that there was generally a lack of agreement on the associations of these medications with the subsequent risk of ALS between studies. However, it appeared that statins, aspirin, OCs/HRT, antihypertensives, and antidiabetics were unlikely related to a higher risk of ALS. The positive associations suggested for the use of antibiotics, antidepressants and skeletal muscle relaxants might be attributable to prodromal symptoms of ALS.

An inverse association of cholesterol lowering drugs with ALS risk was found in four studies [8–11], while a null or marginal association was observed in four [7, 12, 13, 22]. The marginal association was likely due to the higher risk of ALS during the first year after statin use [13]. The differing results between studies might be attributable to diverse study designs, sample sizes, variable adjustments, or biological difference between populations. Although still controversial, some studies have suggested that hyperlipidemia may be a risk factor ALS [31, 32]. If this is the case, the null or inverse association noted between cholesterol lowering drugs, especially statins, and ALS risk might indicate a potentially protective role of statins on ALS. After considering indications for statin use, Freedman et al. indeed found a lower ALS risk in relation to statin use, but not other cholesterol-lowering drugs [10]. The potentially protective role of statins may be attributed to their antioxidative and anti-inflammatory properties [33].

Steroids were not associated with the risk of ALS in the present study. Some experimental studies have shown that steroids may exhibit a neuroprotective role on neurons, glial cells, and blood vessels via steroid receptor signaling [34, 35], whereas others found a detrimental effect of steroids on motor neurons [36–38]. In terms of NSAIDs, most previous studies failed to show an association with ALS risk [14–16], except for one [22]. The discrepancy between studies might be partly due to sample size, recall bias with self-reported data, not considering reverse causation, and lack of correction for multiple testing. Real population differences might also exist. For instance, two US studies did not find an association between aspirin use and ALS [14, 15], whereas two Taiwanese studies found aspirin use to be associated with a lower risk of ALS [16]. A potentially protective role of aspirin on ALS has been identified in an experimental study which suggested that a derivative of aspirin “AAD-2004” might decrease motor neuron degeneration and improve motor function and life span by blocking free radical production, prostaglandin E [2] formation, and microglial activation in the spinal cord of ALS mice [39].

The findings on immunosuppressants are inconsistent [9, 17, 22]. Further, immunosuppressive treatment was not shown to be related to disease progression and survival of ALS patients [40, 41]. Emerging evidence suggests that gut microbiota dysbiosis might influence the development and disease progression of ALS through the microbiota-gut-brain axis [42, 43]. The relationship between antibiotics use and ALS risk, however, is less studied in human. One study showed a positive association, which was mainly attributable to the use of antibiotics during the years before ALS diagnosis [18].

There is similarly no agreement on the association of OCs or HRT use with the risk of ALS, but findings point to a lowered or null risk. OCs use differs greatly between populations due to cultural and socioeconomic differences [21] and is associated with many factors such as age, marital status, and health status [44, 45]. Such differences may have contributed to the difference in findings of different studies. In animal and cell studies, however, estrogen was found to exert neuroprotective effects on motor neurons through binding estrogen receptor α [46, 47] and hinder the progression of the female ovariectomized *SOD1*^*G93A*^ mice. The prognostic role of estrogen has also been studied in ALS patients, however, with no considerable influence on disease progression [22].

In general, antihypertensive drugs were not shown to be associated with the risk of ALS. An Italian study demonstrated null associations of ACEIs and ARBs with the risk of MND [25], which differed from the finding of a protective role of ACEIs on ALS risk in a Taiwanese study [16]. The ethnic and age differences between the studies might explain the discrepancy to some extent. Further, the disparity in vitamin E exposure between the two populations may also contribute as ACEIs may function through increasing the levels of vitamin E whereas a protective role of ACEIs might disappear at the presence of relatively high vitamin E levels [25].

Antidiabetics use was shown to be associated with a lower risk of ALS, although two studies did not find statistically significant results [8, 22] which might be partly attributed to their relatively small sample sizes. It is difficult to conclude a neuroprotective role of antidiabetics on development of ALS due to the difficulty of discerning antidiabetics use from underlying diabetes. Previous studies have indeed found type 2 diabetes to be associated with a lower risk of ALS [48, 49]. A recent study has further suggested that the relationship might be causal in the European population [49]. Experimental studies have also supported a protective role of antidiabetics on ALS development as ALS mice treated with pioglitazone exhibited delayed onset and prolonged survival, in parallel with morphological and functional improvement of motor neurons [50].

Antidepressants and a skeletal muscle relaxant, baclofen, were found to be associated with a higher risk of ALS. Reverse causation might be the main explanation for these associations as depression and muscle problems are also symptoms of ALS and the risk is decreasing with longer time before diagnosis [6, 22, 28]. For instance, patients with ALS were more likely to receive a diagnosis of depression before diagnosis of ALS, especially during the year before ALS diagnosis [28]. Although a higher ALS risk was suggested in relation to antiepileptics use in one study [30], the association was not statistically significant. No genetic correlation was observed between ALS and epilepsy either [51]. Opioids and lithium, on the other hand, were found to be associated with a lower risk of ALS. Opioids exert neuroprotective effect through stimulating opioid receptors and upregulating excitatory amino acid transporters [52]. The protective effect of lithium in ALS may occur through an activation of autophagy in CNS [53]. Besides, findings that ALS patients with the *UNC13A C/C* genotype might benefit from lithium treatment suggesting a role of lithium in the promotion of synaptogenesis and neuronal outgrowth [54]. However, as these medications have been relatively rarely studied so far, a conclusion cannot be drawn before more studies are available.

To our knowledge, this is the first systematic review to evaluate the association between medication use and subsequent risk of ALS. The strengths of this review include the systematic search strategy using three large databases and the large numbers of articles included without restricting the publishing time. Further, the quality of the included studies was moderate to high according to the results of the quality assessment tool NOS.

Nonetheless, this review has several limitations. First, variation exists between studies regarding genetic background, ethnicity, geographic location, sample size, study design, consideration of reverse causation, and multivariable adjustment, making it difficult to make direct comparison between studies. Specifically, as ALS likely has a long pre-clinical stage with various prodromal symptoms and there is a known diagnostic delay of about 1 year for ALS, due to patient and healthcare-related delays [55], studies not considering potential reverse causation due to such might reach artificial conclusions regarding beneficial or harmful medications. Regardless, similar findings across different genetic background, ethnicity, and geographic location may on the other hand provide a good opportunity to find a robust risk factor for ALS. Second, some medications were only studied as a broad category, leaving the drug-specific effect unraveled. Third, some studies were based on patients with other diseases such as psychiatric disorders [29] and non-melanoma skin cancer [56], which may lead to low generalizability of the results to the general population. Finally, we focused on articles in English in three large databases (Medline, Embase, and Web of Science), studies from other databases or in other languages were not included. The number of the latter is however presumably small.

The increased availability of medication data obtained from medical records and administrative databases has greatly promoted the development of pharmaco-epidemiological research [57]. Although there are inherent challenges in pharmaco-epidemiological studies, namely that it is difficult to disentangle the effect of the studied medication from its underlying disease indication, so-called confounding by indication or indication bias, pharmaco-epidemiological studies are currently the main research tool to evaluate the long-term benefits and adverse events of medication use in the real-world practice [58]. Indication bias might be partially addressed by specific study designs, such as restricting the analysis to a target population (e.g., patients with specific disease) with or without medication use and active comparator design (i.e., comparing the effect of the target medication with another active drug) [59]. These designs also have limitations. For instance, they often rely on the assumption that there is little difference between the comparison groups other than medication use; however, it is almost always true that treated and untreated individuals as well as patients with different treatments for the same disease differ in disease severity, comorbidity profiles, etc.

As previous reviews have summarized the existing literature on the roles of specific diseases, including cardiometabolic and inflammatory diseases, on the risk of ALS [60, 61], in the present review, we focused on medication use as potential risk factor for ALS. However, because among the studies included in the present review, few have made efforts to address the concern of indication bias, it remains largely unknown whether it is the use of a specific medication or its underlying disease that is the real relevant factor for ALS. Although indication bias is unlikely to be completely ruled out in any pharmaco-epidemiological studies to date, new efforts should regardless be encouraged to use state-of-the-art study designs, such as analysis of target population [62], use of active comparator [63], application of propensity score to match comparison groups [64], and target trial emulation [65], to estimate the potential influence of indication bias. It would also be useful to provide more information, if available, on medication use, including indication for use, specific types of medication instead of using only a broad category of the medication, and dose and quantity of medication use.

In addition to indication bias, as important potential confounders such as smoking are rarely available in previous studies, residual confounding due to factors other than indication can also be a common source of systemic error. Further, the low incidence of ALS makes some studies underpowered to disclose real associations with relatively small magnitude from noise. Finally, the heterogeneity of ALS as a disease means that an overall null association for ALS in general does not necessarily preclude an association for a specific type of ALS and this has rarely been addressed in the literature.

Finally, the present review focused on studies where different medications were studied as potential risk factors for ALS. Additional efforts should be made to summarize the existing literature where medications are studied as potential factors influencing prognosis of ALS. For instance, one study suggested an association between status use and functional decline among ALS patients [66], whereas another study failed to do so [67]. In another study, aspirin or other NSAIDs use was found to be associated with a shorter survival among ALS patients; however, the association disappeared after correcting for multiple testing [68]. Similarly, although animal and cell studies have found estrogen to exert neuroprotective effect on motor neurons [46, 47], human studies failed to show an influence of estrogen on disease progression of ALS [22]. Finally, in terms of antidiabetics, a randomized trial evaluated pioglitazone as an add-on therapy in ALS patients without however showing beneficial effects on survival [69].

## Conclusions

Although there is currently no strong evidence to link any medications with a higher risk of ALS, it appears that statins, aspirin, OCs/HRT, antihypertensives, and antidiabetics were unlikely related to a higher risk of ALS. Although this might provide evidence for the continued use of these medications among individuals at high-risk of ALS, interpretation of these results should be done with caution as most of the studies examined broad categories of these medications, leaving the potentially harmful role of specific drug classes unraveled. The positive association noted for antibiotics, antidepressants, and skeletal muscle relaxants might on the other hand be attributable to prodromal ALS symptoms or reverse causation due to diagnostic delay.

## Supplementary Information

Additional file 1. Documentation of searchstrategies.

## Data Availability

Not applicable.

## Abbreviations

ALS: Amyotrophic lateral sclerosis
NOS: Newcastle-Ottawa Assessment Scale
OCs: Oral contraceptives
HRT: Hormone replacement therapy
PRISMA: Preferred Reporting of Systematic Reviews and Meta Analyses
MND: Motor neuron disease
COPD: Chronic obstructive pulmonary disease
NSAID: Non-steroidal anti-inflammatory drug
ACEIs: Angiotensin-converting enzyme inhibitors
ARBs: Angiotensin II receptors blockers
cDDD: Cumulative defined daily dose
PPI: Proton pump inhibitor
CNS: Central nervous system
ADHD: Attention deficit hyperactivity disorder
