# Supplementary material for: Medication use and risk of amyotrophic lateral sclerosis—a systematic review
Source: BMC Med. 2022 Aug 5;20:251. doi: 10.1186/s12916-022-02442-w (PMC9354307; doi:10.1186/s12916-022-02442-w)
Supplement: Supplementary file 1 — Documentation of searchstrategies. [file 12916_2022_2442_MOESM1_ESM.docx]

Documentation of search strategies

University Library search consultation group

Date: 10 December 2021

Topic/research question: The association between medication and risk of amyotrophic lateral sclerosis

Name of researcher(s): Can Cui

Librarian(s): Sabina Gillsund & GunBrit Knutssön

Databases:

1. Medline (Ovid)
2. Embase (embase.com)
3. Web of Science

Total number of hits:

- Before deduplication: 4,760
- After deduplication: 3,360

1. Medline

| Interface: Ovid MEDLINE(R) and Epub Ahead of Print, In-Process & Other Non-Indexed Citations and Daily  Date of Search: 10 December 2021  Number of hits: 1,423  Comment: In Ovid, two or more words are automatically searched as phrases; i.e. no quotation marks are needed | Field labels   - exp/ = exploded MeSH term - / = non exploded MeSH term - .ti,ab,kf. = title, abstract and author keywords - adjx = within x words, regardless of order - * = truncation of word for alternate endings |
| --- | --- |
| Database(s): **Ovid MEDLINE(R) and Epub Ahead of Print, In-Process & Other Non-Indexed Citations and Daily**1946 to December 10, 2021 Search Strategy:   \| **#** \| **Searches** \| \| --- \| --- \| \| 1 \| Amyotrophic Lateral Sclerosis/ci \| \| 2 \| Amyotrophic Lateral Sclerosis/ \| \| 3 \| Motor Neuron Disease/ \| \| 4 \| (amyotrophic lateral scleros?s or charcot disease* or gehrig* disease* or guam disease* or motor neuron disease*).ti,ab,kf. \| \| 5 \| 2 or 3 or 4 \| \| 6 \| exp Pharmacologic Actions/ \| \| 7 \| exp Pharmaceutical Preparations/ \| \| 8 \| (activato? or agent? or agonist? or antagonist? or drug? or inducer? or inhibitor? or medication? or medicine? or modulator? or pharmaceutic* or pharmacolog* or reactivator? or substance?).ti,ab,kf. \| \| 9 \| 6 or 7 or 8 \| \| 10 \| exp Causality/ \| \| 11 \| Incidence/ \| \| 12 \| Risk/ \| \| 13 \| Dose-Response Relationship, Drug/ \| \| 14 \| exp Product Surveillance, Postmarketing/ \| \| 15 \| (adverse drug reaction reporting* or adverse event reporting* or drug? surveillance or pharmaco-vigilance* or pharmacovigilance*).ti,ab,kf. \| \| 16 \| ((postmarket* or post-market*) adj2 (data or evaluation or study or studies or surveillance)).ti,ab,kf. \| \| 17 \| (dose-response or incidence or risk).ti,ab,kf. \| \| 18 \| ((etiology or caus* or develop*) adj5 (ALS or amyotrophic lateral scleros?s or charcot disease* or gehrig* disease* or guam disease* or motor neuron disease*)).ti,ab,kf. \| \| 19 \| or/10-18 \| \| 20 \| 5 and 9 and 19 \| \| 21 \| 1 or 20 \| \| 22 \| exp Clinical Trial/ \| \| 23 \| exp Review/ \| \| 24 \| Comment/ \| \| 25 \| Editorial/ \| \| 26 \| Case Reports/ \| \| 27 \| or/22-26 \| \| 28 \| 21 not 27 \| \| 29 \| 28 not (animals not humans).sh. \| \| 30 \| limit 29 to english \| | |

2. Embase

| Interface: embase.com  Date of Search: 10 December 2021  Number of hits: 2,495  Comment: Emtree is the controlled vocabulary in Embase | Field labels   - /exp = exploded Emtree term - /de = non exploded Emtree term - ti,ab,kw = title, abstract and author keywords - NEAR/x = within x words, regardless of order - * = truncation of word for alternate endings |
| --- | --- |
| **No.**  **Query**  **#29**  **#28** NOT ([animals]/lim NOT [humans]/lim) AND [english]/lim  **#28**  **#27** AND (**'article'**/it OR **'article in press'**/it OR **'erratum'**/it)  **#27**  **#21** NOT **#26**  **#26**  **#22** OR **#23** OR **#24** OR **#25**  **#25**  **'case report'**/exp  **#24**  **'editorial'**/exp  **#23**  **'review'**/exp  **#22**  **'clinical trial'**/exp  **#21**  **#4** AND **#8** AND **#20**  **#20**  **#9** OR **#10** OR **#11** OR **#12** OR **#13** OR **#14** OR **#15** OR **#16** OR **#17** OR **#18** OR **#19**  **#19**  ((**postmarket*** OR **'post-market*'**) NEAR/2 (**data** OR **evaluation** OR **study** OR **studies** OR **surveillance**)):ti,ab,kw  **#18**  **'adverse drug reaction reporting*'**:ti,ab,kw OR **'adverse event reporting*'**:ti,ab,kw OR **'drug? surveillance'**:ti,ab,kw OR **'pharmaco-vigilance*'**:ti,ab,kw OR **pharmacovigilance***:ti,ab,kw  **#17**  ((**etiology** OR **caus*** OR **develop***) NEAR/5 (**als** OR **'amyotrophic lateral scleros?s'** OR **'charcot disease*'** OR **'gehrig* disease*'** OR **'guam disease'** OR **'motor neuron disease*'**)):ti,ab,kw  **#16**  **'dose-response'**:ti,ab,kw OR **incidence**:ti,ab,kw OR **risk**:ti,ab,kw  **#15**  **'postmarketing surveillance'**/exp  **#14**  **'pharmacovigilance'**/exp  **#13**  **'dose response'**/exp  **#12**  **'incidence'**/de  **#11**  **'risk factor'**/exp  **#10**  **'risk'**/de  **#9**  **'causality'**/de  **#8**  **#5** OR **#6** OR **#7**  **#7**  activato$:ti,ab,kw OR agent$:ti,ab,kw OR agonist$:ti,ab,kw OR antagonist$:ti,ab,kw OR drug$:ti,ab,kw OR inducer$:ti,ab,kw OR inhibitor$:ti,ab,kw OR medication$:ti,ab,kw OR medicine$:ti,ab,kw OR modulator$:ti,ab,kw OR **pharmaceutic***:ti,ab,kw OR **pharmacolog***:ti,ab,kw OR reactivator$:ti,ab,kw OR substance$:ti,ab,kw  **#6**  **'chemicals and drugs'**/exp  **#5**  **'drug mechanism'**/exp OR **'drug exposure'**/exp  **#4**  **#1** OR **#2** OR **#3**  **#3**  'amyotrophic lateral scleros$s':ti,ab,kw OR **'charcot disease*'**:ti,ab,kw OR **'gehrig* disease*'**:ti,ab,kw OR **'guam disease*'**:ti,ab,kw OR **'motor neuron disease*'**:ti,ab,kw  **#2**  **'motor neuron disease'**/de  **#1**  **'amyotrophic lateral sclerosis'**/exp | |

3. Web of Science Core Collection

| Interface: Clarivate Analytics  Date of Search: 10 December 2021  Number of hits: 842 | Field labels   - TS/Topic = title, abstract, author keywords and Keywords Plus - NEAR/x = within x words, regardless of order - * = truncation of word for alternate endings   Note: sometimes “quotation marks” are needed for single search terms to avoid automatic term mapping (lemmatization). |
| --- | --- |
| #1 TS=("amyotrophic lateral scleros?s" or "charcot disease*" or "gehrig* disease*" or "guam disease*" or "motor neuron disease*")  #2 TS=(activato$ or agent$ or agonist$ or antagonist$ or drug$ or inducer$ or inhibitor$ or medication$ or medicine$ or modulator$ or pharmaceutic* or pharmacolog* or reactivator$ or substance$)  #3 TS=(("dose-response" or "incidence" or "risk")) OR TS=(((etiology OR caus* OR develop*) NEAR/4 (“als” OR "amyotrophic lateral scleros?s" OR "charcot disease*" OR"'gehrig* disease*" OR "guam disease" OR "motor neuron disease*"))) OR TS=(("adverse drug reaction reporting*" or "adverse event reporting*" or "drug? surveillance" or "pharmaco-vigilance*" or pharmacovigilance*)) OR TS=(((postmarket* or "post-market*") NEAR/1 (data or evaluation or study or studies or surveillance)))  #4 #3 AND #2 AND #1  Refined by: DOCUMENT TYPES: ( ARTICLE OR EARLY ACCESS ) AND LANGUAGES: ( ENGLISH ) | |
